# Gilgamesh is required for the maintenance of germline stem cells in *Drosophila* testis

**DOI:** 10.1038/s41598-017-05975-w

**Published:** 2017-07-18

**Authors:** Dongsheng Chen, Xiangxiang Zhu, Lijuan Zhou, Jian Wang, Xiaoqian Tao, Shuang Wang, Fuling Sun, Xianzhao Kan, Zhengqi Han, Yuelin Gu

**Affiliations:** 1 Provincial Key Laboratory of the Conservation and Exploitation Research of Biological Resources in Anhui, College of Life Sciences, Anhui Normal University, Wuhu, 241000 China; 2 The Institute of Bioinformatics, College of Life Sciences, Anhui Normal University, Wuhu, 241000 China

## Abstract

Emerging evidence supports that stem cells are regulated by both intrinsic and extrinsic mechanisms. However, factors that determine the fate of stem cells remain incompletely understood. The *Drosophila* testis provides an exclusive powerful model in searching for potential important regulatory factors and their underlying mechanisms for controlling the fate of germline stem cells (GSCs). In this study, we have found that *Drosophila gilgamesh* (*gish*), which encodes a homologue of human CK1-γ (casein kinase 1-gamma), is required intrinsically for GSC maintenance. Our genetic analyses indicate *gish* is not required for Dpp/Gbb signaling silencing of *bam* and is dispensable for Dpp/Gbb signaling-dependent Dad expression. Finally, we show that overexpression of *gish* fail to dramatically increase the number of GSCs. These findings demonstrate that *gish* controls the fate of GSCs in *Drosophila* testis by a novel Dpp/Gbb signaling-independent pathway.

## Introduction

Adult stem cells (ASCs) are essential for tissue homeostasis by constantly providing new cells to replenish many tissues, including blood, skin, germ-line, and the intestinal epithelium. ASCs are characterized by self-renewal and potentiality to differentiation during all life time, and a balance between self-renewal and differentiation is crucial for tissue homeostasis^1–3^. Previous studies have shown that the intrinsic factors from stem cells are necessary to achieve this balance, extrinsic signaling molecules from microenvironment (also called “niche”) surrounding ASCs also control this balance^4–6^.

**Figure 1:**
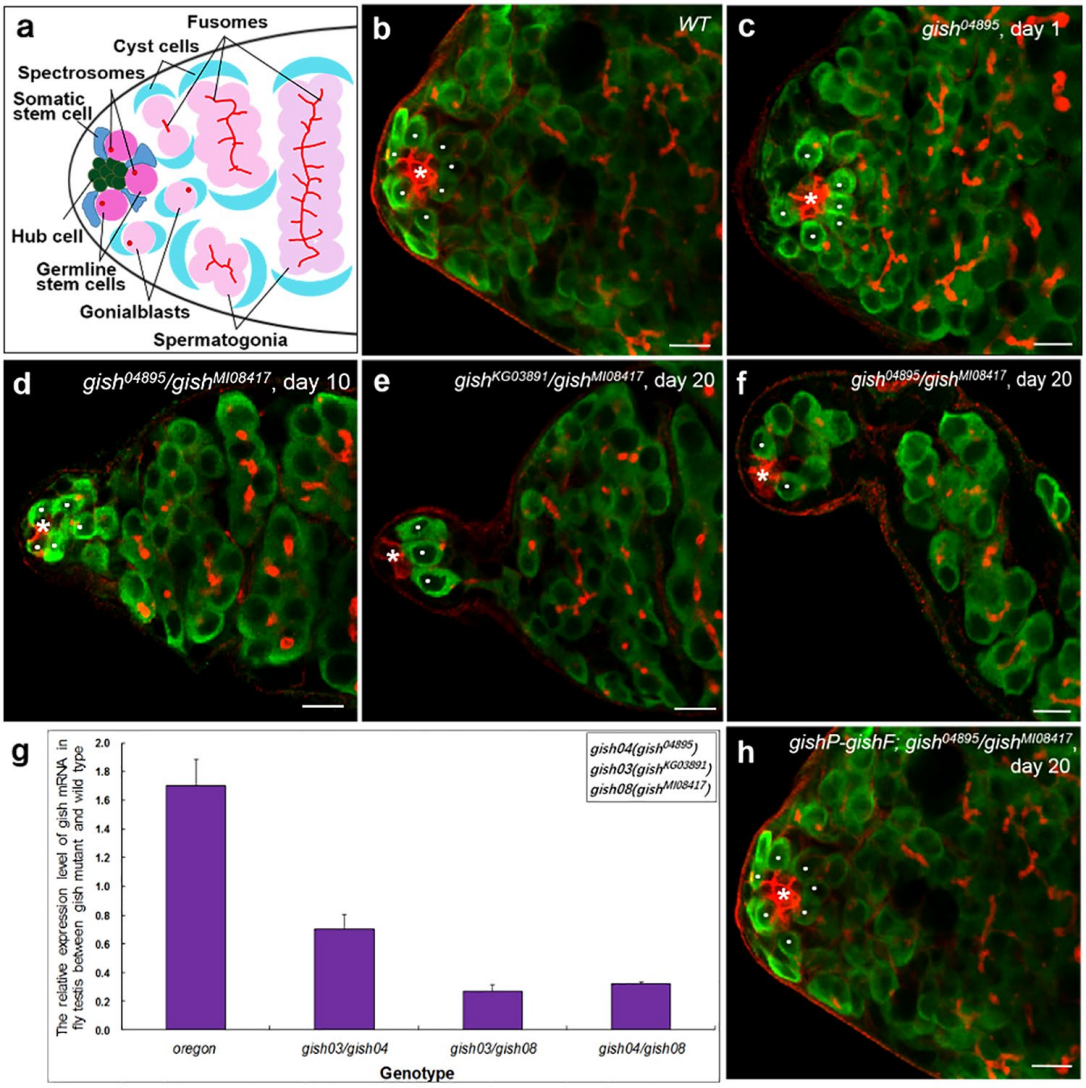
Identification of a *gish* mutant with defects in male GSCs maintenance. (**a**) A diagram of a testis tip with different cell types labeled with different colors: Germline stem cells (GSCs) (dark pink), Hub cell (green), Somatic stem cell (SSC) (dark blue), Gonialblasts (GBs) (pink), cyst cells (blue), and fusomes (red). (**b**–**f**,**h**) Testes stained with anti-Fas III antibody to label the hubs (red, indicated by asterisks), anti-Hts antibody to label the fusomes (red), and anti-Vasa antibody to label germ cells (green). GSCs were highlighted by white dots. (**b**) In wild-type testis, seven GSCs (white dot) directly contact the hub (asterisk). (**c**) *gish* ^04895^ mutant testis from 1-day-old fly showing that six GSCs contact the hub. (**d**) *gish* ^04895^/*gish* ^MI08417^ testis from 10-day-old fly showing that five GSCs remains adjacent to the hub. (**e**) *gish* ^KG03891^/*gish* ^MI08417^testis from 20-day-old fly showing three remaining GSCs. (**f**) *gish* ^04895^/*gish* ^MI08417^ testis from 20-day-old fly. Only two GSCs remains. (**g**) Quantitative PCR analyses of *gish* mRNA levels in testes between *gish* mutant and wild-type. (**h**) The transgene P{*gishP-gishF*} rescued the *gish* ^04895^/*gish* ^MI08417^ male testis to normal, with eight GSCs close to the hub. Scale bars: 10 μm.

Little is known for the mechanisms of stem cell regulation which maintains this balance between self-renewal and differentiation. The *Drosophila* testis provides an exemplary model for the study of stem cell biology^7, 8^. In adult *Drosophila* males, two stem cell populations are located at the apical tip of the testis: germline stem cells (GSCs) and somatic stem cells (SSCs) (Fig. 1a). Both GSCs and SSCs contact with a cluster of non-dividing somatic cells known as the hub. A GSC divides asymmetrically to generate one daughter cell, which maintains adjacent to the hub and retains stem cell identity, and the other one, which is pushed away from the hub and initiates differentiation as a gonialblast (GB). The GB mitotically divides four times to produce a cyst of 16 interconnected spermatogonia, which go on to enter meiosis and differentiate into spermatid, eventually maturing into sperms. During the process of GSCs dividing, the hub functions as a major component of GSCs niche. SSCs serve both as another component of GSCs niche and as stem cells to generate cyst cells (CCs) which encapsulates the differentiating GBs^9^.

The casein kinase 1 (CK1) family is evolutionarily conserved from yeast to human, and regulates multiple physiological processes, such as membrane transport, circadian rhythm, apoptosis, vesicle transport, cell division and differentiation^10^. *Drosophila gilgamesh* (*gish*), which encodes a homologue of human CK1-γ (casein kinase 1-γ), has been shown to be involved in glial cell migration, olfactory learning, spermatogenesis and the Wg/Wnt pathway^11–16^. Here, we identified a new role for *gish* in maintaining the fate of germline stem cells in male *Drosophila*.

## Results

### Identification of *gish* mutants with defects in GSC maintenance

To identify novel genes that regulate the self-renewal or differentiation of GSCs in *Drosophila* testis, we conducted a screen for male sterile mutants with P-element insertion, available from the Bloomington Stock Center. We isolated a line with a P-element insertion in the third chromosome^15^, P{PZ}*gish* ^04895^, which resulted in small testis and reduction in germ-cell number in homozygous mutant males. To thoroughly analyze the behavior of germ cells in *gish* ^04895^ mutant, we used anti-Fas III and anti-Vasa antibodies to visualize hub and germ cells^17, 18^, respectively (Fig. 1b). Hub is a cluster of somatic cells found at the tip of the adult testis which could be characterized by anti-Fas III antibody staining in *Drosophila*. As shown in Fig. 1b, we visualized GSCs and germ cells with an anti-Hts antibody^19^. A spherical fusome (also called the “spectrosome”) is the marker of GSCs and GB. GB undergoes 4 rounds of cell division to produce a 16-cell germline cluster, in which branched fusomes are visualized by the anti-Hts antibody (Fig. 1a,b).

In the tip of wild-type testis, 6–10 GSCs that were directly adjacent to the hub were recognized by an anti-Vasa antibody (Fig. 1b)^17^. In contrast, in the 10-day-old testes from *gish* ^04895^ homozygous males, about 30–40% of mutant testes (n > 100) contained 3–4 GSCs attached to the hub. This finding suggests that *gish* may plays a key role in the maintenance of GSCs. To explore whether *gish* is involved in the GSC maintenance in other relevant genetic background, we next performed phenotypic analyses with *gish* removal in three allelic combinations (Table 1). Using the methods described in the previous study^17^, we counted the number of GSCs in *gish* allelic combinations at days 1, 10 and 20 after eclosion. Compared to wild-type and *gish*/+ heterozygotes, the newly eclosed *gish* mutants (*gish* ^04895^/*gish* ^KG03891^, *gish* ^04895^/*gish* ^MI08417^, *gish* ^KG03891^/*gish* ^MI08417^) contained an average of 6.7, 6.5 and 6.3 GSCs/testis (Fig. 1c and Table 1). When these three *gish* mutants were cultured at room temperature for 10 days, the testes had an average of 5.4, 5.7 and 5.8 GSCs/testis respectively (Fig. 1d and Table 1), whereas the testes from wild-type contained a normal average of 7.7 GSCs/testis. At day 20, the average number of GSCs was dramatically reduced to 3.9, 3.7 and 3.5 GSCs/testis respectively (Fig. 1e,f and Table 1), compared to the average number of wild-type maintained at the normal level (6.0 GSCs/testis) (Table 1). The testes from *gish* ^*04895*^/*Df* and *gish* ^*KG03891*^/*Df* exhibited a similar loss of GSCs with ageing (Table 1). These statistical data indicate that the deficiency of *gish* leads to a progressive loss of GSCs with ageing.

**Table 1:** Phenotypic assay for *gish* mutant flies.

Genotype	The average number of GSCs in fly testis with the elapse of days (Mean ± SD)		
	Day 1	Day 10	Day 20
*Oregon-R*	8.1 ± 1.0 (n = 97)	7.7 ± 1.1 (n = 61)	6.6 ± 0.7 (n = 65)
*gish* ^*04895*^/+	8.1 ± 0.9 (n = 56)	7.5 ± 1.0 (n = 60)	6.7 ± 0.8 (n = 60)^#^
*gish* ^*KG03891*^/+	8.0 ± 1.0 (n = 50)	7.7 ± 0.8 (n = 57)	6.5 ± 0.9 (n = 56)^#^
*gish* ^*MI08417*^/+	8.0 ± 0.8 (n = 60)	7.7 ± 0.9 (n = 55)	6.6 ± 0.8 (n = 66)^#^
*gish* ^*04895*^/*gish* ^*KG03891*^	6.7 ± 0.9 (n = 55)	5.4 ± 1.0 (n = 52)	3.9 ± 1.0 (n = 62)^∗^
*gish* ^*04895*^/*gish* ^*MI08417*^	6.5 ± 1.0 (n = 51)	5.7 ± 0.8 (n = 62)	3.6 ± 0.7 (n = 73)^∗^
*gish* ^*KG03891*^/*gish* ^*MI08417*^	6.3 ± 0.6 (n = 52)	5.8 ± 0.9 (n = 66)	3.4 ± 0.8 (n = 64)^∗^
*gish* ^*04895*^/*Df*	6.7 ± 1.1 (n = 52)	5.2 ± 0.7 (n = 60)	3.3 ± 0.7 (n = 63)^∗^
*gish* ^*KG03891*^/*Df*	6.0 ± 0.7 (n = 55)	5.5 ± 0.6 (n = 62)	3.2 ± 0.8 (n = 67)^∗^

SD, standard deviation. n, Number of testes examined. *Df*, fly deficiency strain for *gish* gene. ^#^ *P* > 0.05; *P < 0.01, unpaired *t*-test, compared with *Oregon-R* at day 20.

To test whether the GSC loss phenotype is due to a reduced *gish* expression level in *gish* mutant testis, we performed real-time quantitative polymerase chain reaction (qPCR) experiments to compare the mRNA level between the wild-type and mutant fly testis^20^. The *gish* gene totally has thirteen predicted mRNA splicing variants (A, B, C, D, E, F, G, H, I, J, K, L and M) derived from the *Drosophila* database (www.flybase.org), but their corresponding encoded proteins share a conserved kinase domain. Based on this, the qPCR primers were designed (see the materials and methods) to target the conserved cDNA region using the online Primer 3.0 version software to detect the whole mRNA expression level of *gish*. We extracted total RNA from *Drosophila* testes, performed reverse-transcription (RT), and conducted qPCR experiments to measure the whole *gish* mRNA level with the rp49 gene as reference^21^. Compared to wild-type, the total *gish* mRNA expression levels in *gish* mutant testes (*gish* ^04895^/*gish* ^KG03891^, *gish* ^KG03891^/*gish* ^MI08417^and *gish* ^04895^/*gish* ^MI08417^) were severely reduced (Fig. 1g). These results strongly suggest that Gish is reduced in these *gish* mutants’ testes, and also imply that the Gish protein is responsible for the loss of GSCs phenotype in *gish* mutant flies.

To further confirm the role of *gish* in GSC maintenance, we next performed a *gish* rescue assay using *gish* cDNA. The *gish* gene contains numerous splicing variants as mentioned above. To explore which isoforms are expressed in *Drosophila* testis, we designed a dozen of primer pairs (Supplementary Table S1), each for each known *gish* transcript, and performed RT-PCR analysis^22^ using total RNAs isolated from wild-type fly testis. We detected transcripts *gishF* and *B* in testis (Supplementary Fig. S1). Based on these findings, we generated a transgene of P{*gishP-gishF*}, in which the *gishF* cDNA was placed under the control of a 5.0 kb *gish* promoter. We found that the GSC loss phenotypes in three *gish* allelic mutants were fully rescued by the transgenic line of P{*gishP-gishF*} (Fig. 1h and Supplementary Table S2). Taken together, our findings demonstrate that the *gish* gene plays an essential role in GSC maintenance.

Previous study has reported that *gish* mutant germ cells contain abnormal actin cones in individualizing spermatid cysts^15^, this result implies that *gish* possibly affects the F-actin-mediated cell adhesion junctions. To explore whether the *gish* mutant GSCs lose adhesion to the hub, we visualized F-actin with phalloidin^15^ and labeled GSCs with anti-Vasa antibody^17^. We found that there was no difference in F-actin-based GSCs adhesion to hub cells between wild-type control and *gish* ^KG03891^/*gish* ^MI08417^ mutant testes (n > 80) at day 14 after eclosion (Supplementary Fig. S2), just as the wild-type, GSCs from *gish* mutant testes contacted directly to hub cells. Similar phenotype was founded in *gish* ^04895^/*gish* ^MI08417^ mutant testes (n > 70). These results manifest that the mutation of *gish* gene doesn’t affect cell-cell (GSC and hub cell) adhesions in fly testes, suggesting some other mechanisms maybe responsible for the GSCs loss phenotype.

To address whether the loss of GSCs in *gish* mutants was due to premature differentiation or cell death of GSCs, we measured the rate of apoptosis in the *gish* mutant GSCs by TUNEL assays^23^. We found that there was no difference in apoptosis between wild-type control and *gish* mutant GSCs (Supplementary Table S3 and Supplementary Fig. S3). These results suggest that mutant GSCs may have deficiency in GSC self-renewal and/or switch to pre-differentiation.

### The *gish* gene is intrinsically required for GSC maintenance

**Figure 2:**
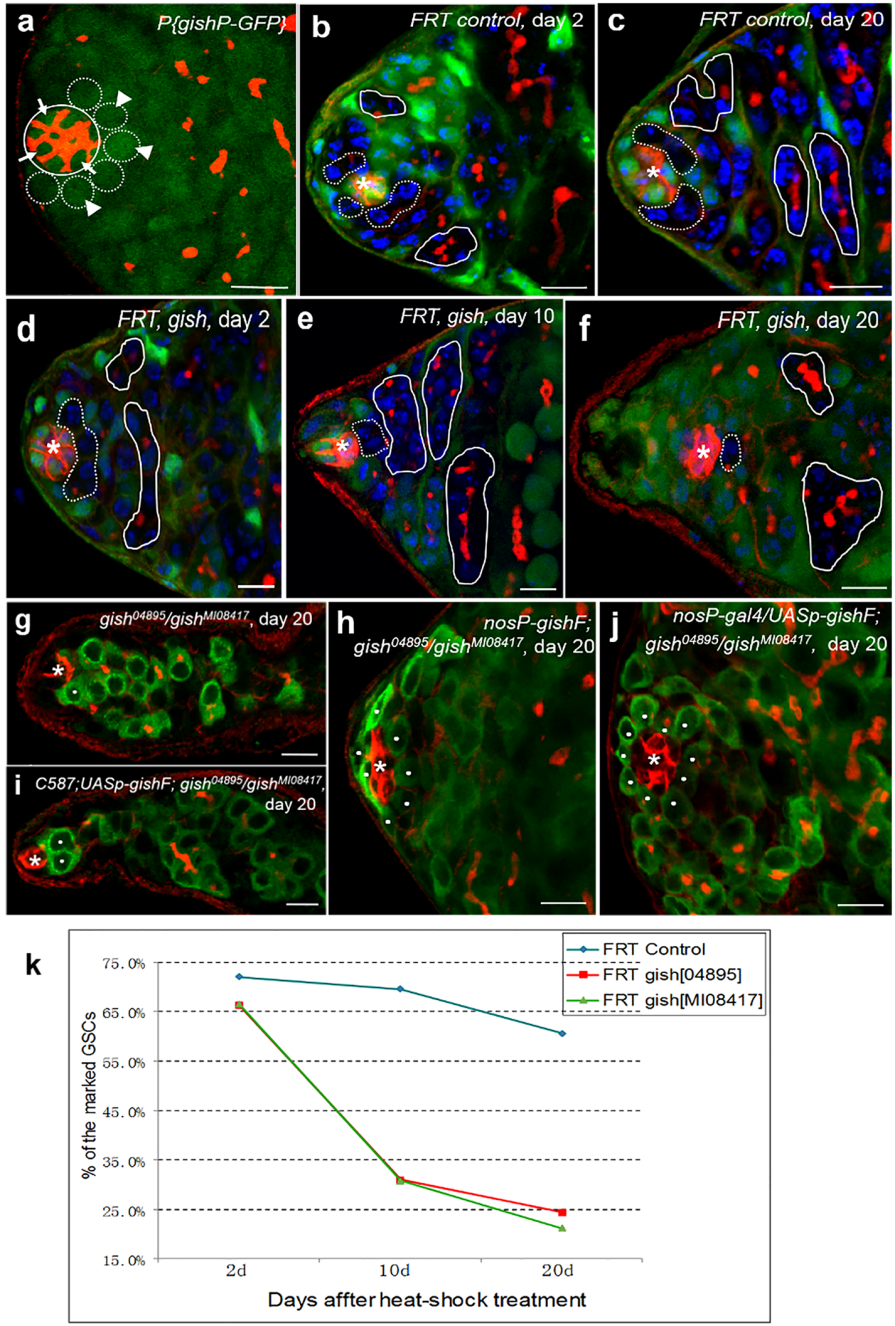
Gish is required intrinsically for GSC maintenance. (**a**) Testis bearing a transgene P{*gishP-GFP*} was stained with anti-Fas III antibody (red) to label the hub (a circle), anti-Hts antibody (red) to visualize fusomes, and anti-GFP antibody (green) to show the *gish* expression pattern. The gene *gish* expresses (green) both in GSCs (broken lines, arrowhead) and in hub cells (a circle, arrow). (**b**–**f**) GSC clones were induced in testes by FLP/FRT-mediated mitotic recombination in adult flies. Testes from *FRT* control flies (**b**,**c**) and *FRT*, *gish* flies (**d**–**f**) were dissected at different days after heat-shock treatment, then stained with anti-GFP (green), anti-Fas III (red) anti-Hts (red) antibodies and DAPI (blue). Hubs were marked by asterisks. GSC clones (indicated by broken lines) and spermatogonium clones (noted by circles) were identified by lack of GFP expression. (**g**–**j**) Testes from 20-day-old flies were stained with anti-Fas III antibody to label the hubs (red, indicated by asterisks), anti-Hts antibody to label the fusomes (red), and anti-Vasa antibody to label germ cells (green). GSCs were highlighted by white dots. (**g**) *gish* ^04895^/*gish* ^MI08417^ testis showing only one GSC remains. (**h**) *nosP-gishF*; *gish* ^04895^/*gish* ^MI08417^ testis showing normal GSCs number (8 GSCs). (**i**) C587; *UASp-gishF*; *gish* ^04895^/*gish* ^MI08417^ testis. Only two GSCs close to the hub. (**j**) *nosP-gal4*; *UASp-gishF*; *gish* ^04895^/*gish* ^MI08417^ testis showing the restored GSC number (10 GSCs). Scale bars: 10 μm. (**k**) Percentage of the negatively-marked GSC clones (lack of GFP expression, GFP-) in *FRT* control and two *FRT*, *gish* alleles at days 2, 10 and 20 after heat-shock treatment. The percentage of the negatively-marked GSCs (GFP-) lacking *gish* was reduced strongly, compared with the negatively-marked GSCs (GFP-) in *FRT* control.

Previous studies have shown that the maintenance of GSCs is regulated through intrinsic and extrinsic signaling pathways in testis^24, 25^. To explore the role of *gish* in GSC maintenance, we generated a transcriptional reporter P{*gishP-GFP*}, in which the *gfp* expression pattern represents that of *gish* gene^26^. As shown by immunofluorescent staining assays (Fig. 2a and Supplementary Fig. S4), GFP was ubiquitously expressed in all cell types including somatic cells (e.g. hub) and germline cells (e.g. GSCs and GBs) in transgenic fly testes (n > 100), suggesting a broad transcription activity of the *gish* promoter.

To determine whether *gish* acts as an intrinsic or extrinsic regulator, we then generated *gish* mutant GSC clones using the FLP/FRT-mediated mitotic recombination technique^27–29^. The *gish* mutant GSCs were negatively-marked after several days of heat-shock treatments. We counted and compared the loss rate of marked GSC clones, as described previously^17, 23^, between the *FRT* control (*hs-flp; FRT82B*/*FRT82B, ubi-gfp*) and the *gish* mutant genotype (*hs-flp; FRT82B, gish*/*FRT82B, ubi-gfp*), at days 2, 10 and 20 after heat-shock treatments (AHST). As shown in Fig. 2, the rates of marked GSC clones from *FRT* control decreased weakly from the initial of 72.0% (n = 254) at day 2 to the last point of 59.9% (n = 197) at day 20 AHST (Fig. 2b,c and k). Only 16.8% of the marked GSCs were lost during the 20-day AHST period. By contrast, under the same experimental conditions, the initial marked *gish* ^04895^ and *gish* ^MI08417^ mutant GSCs clones were 66.6% (n = 213) and 66.4% (n = 188) respectively at day 2 AHST, whereas they reduced to the rates of 21.2% (n = 189) and 24.4% (n = 279) respectively at day 20 AHST (Fig. 2d–f and k). These results revealed that 68.2% and 63.3% of marked *gish* ^04895^ and *gish* ^MI08417^ mutant GSCs clones were lost during the 20-day AHST. Taken together, these findings suggest that *gish* plays an essential role for GSC maintenance through an intrinsic mechanism. To further confirm this point, we generated a transgene, P{*nosP-gishF*}, in which the *gishF* coding sequence was placed under the control of the promoter of the gene *nanos* that possesses a high expression level in germline cells. We found that the GSCs loss phenotype was fully rescued in *gish* mutant flies carrying the P{*nosP-gishF*} transgene (Fig. 2g,h and Supplementary Table S4). This result supports that Gish functions as an intrinsic factor.

To further test whether *gish* also plays an extrinsic role in GSC self-renewal, we generated a new transgenic fly strain carrying P{*UASp-gishF*}, in which the *gishF* coding sequence is under the control of the *UASp* promoter^30^. Using the Gal4-UAS system, we expressed the Gish protein in somatic hub cells and somatic stem cells by the *c587-gal4*-driven *UASp*-*gishF* expression^17^. We found that the GSCs loss phenotype was not rescued in *gish* mutant testes carrying the *c587-gal4* and *UASp*-*gishF* transgenes (Fig. 2i and Supplementary Table S5). We then expressed *gish* specifically in germ cells that carried transgenes of P{*UASp-gish*} and P{*nosP-gal4*}, in which a germline-specific *nanos-gal4* driver can express the target gene under the control of *UASp* promoter^31^. We found that the *gish* phenotype was fully rescued (Fig. 2j and Supplementary Table S6). This result is consistent with the above observation on the rescuing effect of P{*nosP-gishF*}. Taken together, these data strongly indicate that *gish* is an intrinsic factor that regulates GSC self-renewal.

### *gish* is not required for Dpp/Gbb signaling silencing of *bam*

**Figure 3:**
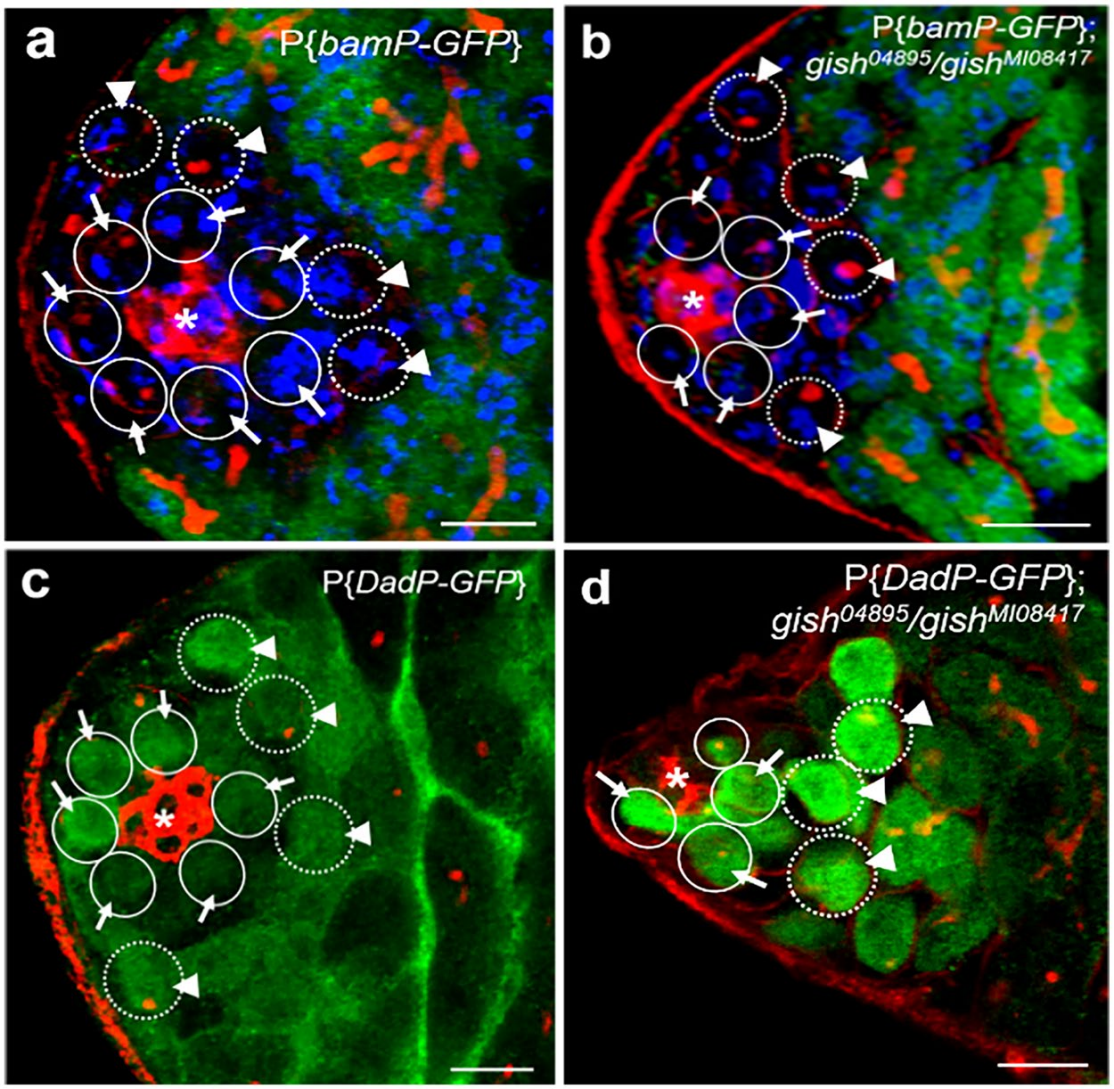
Gish has no affect the expression patterns of *bam* and *Dad*. The testes in (**a**–**d**) were marked with Fas III (red, hub with asterisk), Hts (red, fusomes), GFP (green) and DAPI (blue). GSCs and GBs were highlighted by circles (arrows) and broken lines (arrowheads), respectively. (**a**,**b**) *gish* is not required for Dpp/Gbb signaling silencing of *bam*. Testes from *bamP-gfp* (**a**) and *bamP-gfp; gish* ^04895^/*gish* ^MI08417^ (**b**) male flies show negative GFP expression in either GSCs (indicated by arrows) or GBs (indicated by arrowheads). (**c–d**) *gish* is dispensable for Dpp/Gbb signaling-dependent *Dad* expression. Testes from *DadP-gfp* (**c**) and *DadP-gfp; gish* ^04895^/*gish* ^MI08417^ (**d**) exhibit GFP expression in both GSCs (indicated by arrows) and GBs (indicated by arrowheads). Scale bars: 10 μm.

A Previous study has shown that two BMP members, Dpp and Gbb function cooperatively to maintain GSCs in *Drosophila* testis by repressing *bam* transcription in GSCs^17^. To explore whether *gish* is involved in Dpp/Gbb-dependent *bam* silencing, we examined the *bam* expression pattern in *gish* mutant testes through the expression of *bam* transcriptional reporter P{*bamP-GFP*}^32^. As shown in Fig. 3, the germ cells in testes from 5-day-old flies after eclosion were labelled with anti-GFP antibody and DAPI staining. We found that 88.5% of GSCs and 83% of GBs (n = 65 testes) from the male wild-type flies carrying P{*bamP-GFP*} reporter exhibited a completely negative GFP pattern (Fig. 3a). Similar phenotypes were observed in *gish* mutant testes, 89% of GSCs and 86.5% of GBs (n = 70 testes) showed to be negative for GFP (Fig. 3b). These data strongly suggest that *gish* is not required for *bam* silencing.

### *Gish* is dispensable for *dpp*/*gbb* signaling-dependent *Dad* expression

It has been reported that *dpp* signaling is necessary for *Dad* expression, whereas *Dad* negatively modulates *dpp* signaling, forming a negative-feedback loop in *Drosophila* wing development^33, 34^. Another study shows that *Dad* is a *gbb*-responsive gene that negatively regulates *gbb* signaling in GSCs and GBs in *Drosophila* testis^17^. To test whether mutation of *gish* affects Dpp/Gbb signaling-dependent *Dad* expression in GSCs, we examined the expression of *Dad* transcriptional reporter P{*DadP-GFP*} in the *gish* mutant background. After *gish* mutant males were cultured at 25 °C for five days, 93% of GSCs and 94.5% of GBs (n = 65 testes) of wild-type flies carrying P{*DadP-GFP*} reporter showed positive for GFP (Fig. 3c). Similarly, 95% of GSCs and 90% of GBs (n = 68 testes) from *gish* mutant testes exhibited a GFP-positive pattern (Fig. 3d). These results convincingly suggest that *gish* is dispensable for *Dad* expression.

### Ectopic *gish* expression had no effects on the number of GSC

**Figure 4:**
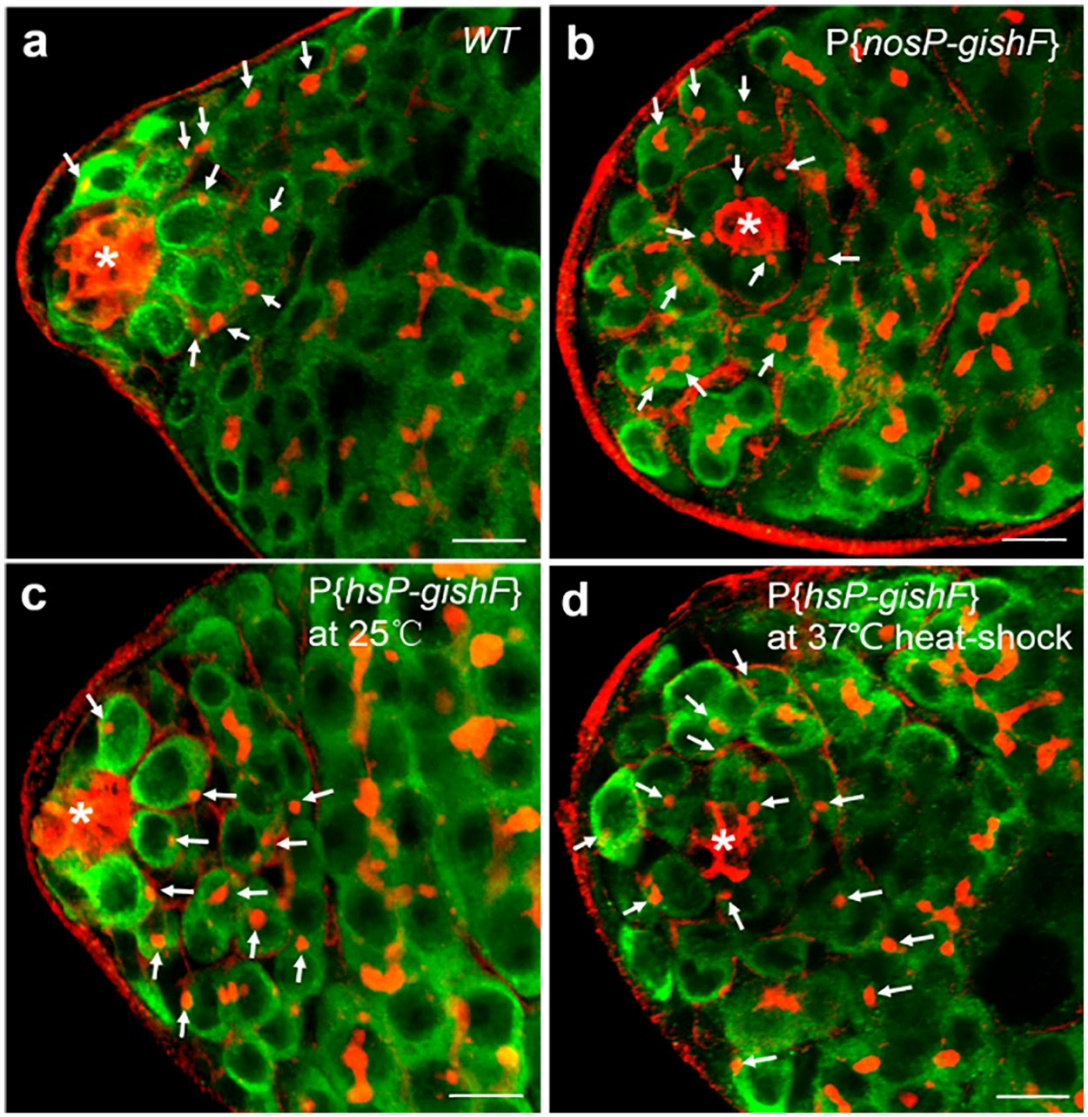
Ectopic *gish* expression had no effect on the number of GSC. The testes in (**a–d**) were stained with anti-Fas III antibody (red, hub with asterisk), anti-Hts antibody (red, fusomes), and anti-Vasa antibody (green, germ cells). (**a**,**b**) Testes were collected from wild-type (**a**) and P{*nosP-gishF*} (**b**) male flies. (**c**,**d**) Testes were dissected from P{*hsP*-*gishF*} male flies that were cultured at 25 °C (**c**) and at 37 °C (**d**) for 1.5 hours three times per day, respectively. Spectrosomes-containing GSCs and GBs are indicated by arrows. Scale bars: 10 μm.

Loss of function of *gish* contributed to testis GSC loss without involvement of apoptosis, it is likely that overexpressed *gish* in testis may delay GSCs/GBs differentiation. To test this possibility, we isolated GSCs from somatic cells and differentiated germ cells by using anti-Hts, anti-fas III and anti-Vasa antibodies to visualize fusomes, hub cells and germ cells, respectively^17, 18^. The spectrosome, a spherical fusome, is the marker of GSCs and their early progeny (also known as GB). When a late-stage GB divides to 2-cell, the fusome is visualized as a short-bar connecting two GB cells. GB undergoes 2, 3 and 4 rounds of synchronous cell division to produce a 4, 8 and 16-cell germline cluster, in which branched fusomes were visualized by anti-Hts antibody (Fig. 1a). We counted the number of germ cells carrying spectrosomes in testes from wild-type, and P{*nosP-gishF*} transgenic flies 7 days after eclosion. We observed the averages of 11.3 spectrosome-contained GSC/GBs (n = 67 testes) per testis in wild-type and 12.5 spectrosome-contained GSC/GBs (n = 70 testes) per testis in P{*nosP-gishF*} flies (Table 2 and Fig. 4a and b). These results suggest that there is no significant difference in the number of spectrosome-contained germ cells among wild-type and *gish*-overexpression transgenic flies.

To substantiate this result, we generated transgenic flies carrying P{*hs-gishF*}, in which a *gishF* cDNA was placed under the control the heat-shock promoter^35^. We overexpressed *gishF* in testes by applying heat-shock treatment at 37 °C for 1.5 hours three times each day, counted the number of germ cells carrying spectromes in testes 7 days after heat-shock treatment with P{*hs-gishF*} flies cultured at 25 °C as control. We found an average of 11.6 GSC/GBs carrying spectrosomes per testis (n = 69 testes) in control flies (Table 2 and Fig. 4c). In contrast, in testes with ectopic *gishF* expression, the number of the germ cells containing spectrosomes was increased to an average of 14.8 cells per testis (n = 72 testes) (Table 2 and Fig. 4d). These results also show that there is no obvious increase in the numbers of spectrosome-containing germ cells after ectopic *gish* expression in P{*hs-gishF*} transgenic flies. Taken together, these data imply that increased Gish activity is not sufficient to block GSC/GB differentiation.

**Table 2:** The statistical analyses of the average number of germ cells carrying spectrosomes in the background of overexpression of *gish*.

Genotype	The average number of germ cells carrying spectrosomes in fly testis with overexpression of *gishF* (Mean ± SD)	*P* Value
*Oregon-R* ^#^	11.3 ± 1.5 (n = 67)	*P* < 0.05
P{*nosP-gishF*}	12.5 ± 1.6 (n = 70)	
P{*hsP*-*gishF*} (RT)^#^	11.6 ± 1.2 (n = 69)	*P* < 0.05
P{*hsP*-*gishF*} (Heatshock at 37 °C)	14.8 ± 1.5 (n = 72)	

^#^The control fly for data analysis. RT, Room temperature. SD, standard deviation. n, Number of testes examined. Unpaired *t*-test, compared with the corresponding control.

## Discussion

Past research has demonstrated that male flies with homozygous *gish* of a P element insertion (P{ry^+t7.2^ = PZ}*gish* ^04895^) exhibited sterile and defective spermatid individualization in *Drosophila* testes^13^. Our previous observation found that the testis became very small in size in *gish* ^04895^ mutant fly. These thinned testes prompted us to explore whether *gish* is involved in the maintenance of germline stem cells in *Drosophila* testis. Combining germline clonal analysis and rescue tests, we showed that *gish* plays an intrinsic role in GSC self-renewal, suggesting that *gish* is necessary to regulate GSCs’ fate. In addition, we found that ectopic *gish* expression only slightly increased the number of GSC-like cells. These results suggest that overexpression of *gish* is not sufficient to repress GSCs/GBs differentiation. Previous studies demonstrated that mutation in *gish* gene led to abnormal nuclei and altered structure of actin cones in the individualizing spermatid cyst^15, 36^, but no further information is available during the early process of GSCs maintenance or switch to differentiation. Our results suggest that *gish* plays a novel role as a GSC intrinsic maintenance factor, but has no roles in the differentiation of GSC/GB in *Drosophila* testis.

Previous research has manifested that Bmp signals from somatic cells, Dpp and Gbb, are essential for the maintenance of GSCs in the *Drosophila* testis^17^. Both Gbb and Dpp function as short-range signals in the tip of the *Drosophila* testis, and their signaling activities are restricted to GSCs and GBs^17, 33, 34^. Interestingly, *Dad* is expressed in GSCs and GBs, whereas *bam* is not expressed in either kind of cell. Both of Dpp and Gbb are essential for maintenance of GSC number^17^. Thus, we checked the *bam* and *Dad* expression pattern using *bam-GFP* and *Dad-GFP* transgene. The results show that the mutation in *gish* has no effect on the expression pattern of *bam* and *Dad* in GSCs and GBs in *Drosophila* testes. These data suggest that *gish* functions downstream of or parallel to *Dad*/*bam*, and is independent of Gbb/Dpp-*bam* signaling pathway.

## Conclusion

In this study, using genetic strategies, we identified and characterized that *Drosophila gish*, encoding a casein kinase 1 protein, as a key player in the regulation of GSCs’ fate. Our results from FLP/FRT-mediated mitotic recombination analyses and rescue assays demonstrate that *gish* functions as an intrinsic factor for maintenance of the number of GSCs in *Drosophila* testis. The action of Gish is dispensable for *bam* silencing or the expression pattern of *Dad*. Our results reveal a new role for the casein kinase 1 (CK1) protein in the fate determination of stem cells.

## Materials and Methods

### *Drosophila* stocks

*Oregon-R* was used as a wild-type strain. The *w* ^*1118*^ strain was used as the host for all P-element-mediated transformations^37^. The following strains were also used for experimentation: (1) *gish* ^04895^, *gish* ^MI08417^ and *gish* ^KG03891^ alleles (Bloomington Stock Center); (2) P{*bamP-GFP*}^38^; (3) P{*nosP-gal4*}, which was described previously^31^. P{*dadP-GFP*}, *neoFRT82B*/*TM3* and *hs-FLP*; *FRT82B, Ubi-GFP*/*TM*_*3*_ was a generous gift from Dr. Dahua Chen. Fly stocks used in this study were maintained at room temperature on a standard medium.

### Histochemistry and microscopy

Testes were prepared for immunostaining as previously described^17, 23^. Primary antibodies were diluted as follows: rabbit anti-GFP (1:500, Invitrogen); mouse monoclonal anti-Hts antibody (1:100, DSHB); rabbit anti-Vasa (1:500, Santa Cruz). Secondary antibodies goat anti-rabbit Alexa 488; goat anti-mouse Alexa 555 (Molecular Probes) were used at a 1:1000 dilution. FITC-conjugated Phalloidin (1:200, Beyotime) was used to visualize F-actin. All samples were examined using a Leica fluorescent microscope, and micrographs were taken using an Olympus confocal FV3000 microscope.

### Generation and Analysis of GSC Clones

Mutant GSC Clones were generated by FLP/FRT-mediated mitotic recombination, as described previously^28, 39^. To generate stocks for stem cell clonal analyses, males of *hs-FLP*; *FRT82B,Ubi-GFP*/*FRT82B*,*gish* ^04895^ and *hs-FLP; FRT82B*,*Ubi-GFP*/*FRT82B*,*gish* ^MI08417^ genotypes (*hs-FLP*; *FRT82B, Ubi-GFP*/*FRT82B* as the wild-type control) were produced by standard genetic crosses. 2-day-old adult males were heat-shocked for 60 minutes at 37 °C three times per day. Five days after heat-shock treatment, testes were dissected for antibody staining at days 2, 10, 20 after last heat-shock treatment. GSC clones were identified and quantified by the lack of GFP expression, as well as their attachment position to the hub cells.

### Detecting gene expression in *Drosophila* testis using RT-PCR and qPCR

Total RNA was extracted from wild-type fly testes using Trizol reagent (Sangon), then the amount of 1 µg was incubated with PrimeScript RTase(50 U/µl) to transcribe cDNA, which was used as the template in PCR reactions, according to the manufactures’ protocol (PrimeScript High Fidelity RT-PCR Kit, Takara). The PCR primers were designed to explore the different *gish* mRNA splicing variants (S1 Table). Total RNAs of fly testes were independently isolated from each phenotype (wild-type and *gish* mutant) using the Trizol reagent (Sangon) and reverse transcribed into cDNA according to the manufactures’ protocol (PrimeScript RT reagent Kit with gDNA Eraser, Takara). For each independent cDNA sample, quantitative PCR was run on CFX96 Touch (BioRad) to measure total *gish* mRNAs with *rp49* as reference according to the manufactures’ protocol (SYBR Premix EX Taq^TM^ II qPCR Kit, Takara). The following primers were used in this study: *gish*, 5′-GCCGGTGGTAAAAGCTCAAG-3′ (sense) and 5′-CGCCAAAATTACCACAGCCA-3′ (antisense); *rp49*, 5′-CACTTCATCCGCCACCAGTC-3′ (sense) and 5′-CGCTTGTTCG ATCCGTAACC-3′ (antisense).

## Electronic supplementary material

Gilgamesh is required for the maintenance of germline stem cells in Drosophila testis

## References

[1] Weissman IL (2000). Stem cells: units of development, units of regeneration, and units in evolution. cell.

[2] Kørbling M, Estrov Z (2003). Adult stem cells for tissue repair - a new therapeutic concept?. New England Journal of Medicine.

[3] Barrilleaux B, Phinney DG, Prockop DJ, O’Connor KC (2006). Review: *ex vivo* engineering of living tissues with adult stem cells. Tissue Engineering.

[4] Morrison SJ, Shah NM, Anderson DJ (1997). Regulatory Mechanisms in Stem Cell Biology. Cell.

[5] Xie T, Spradling AC (2000). A niche maintaining germ line stem cells in the Drosophila ovary. Science.

[6] Allan, S., Fuller, M. T., Braun, R. E. & Shosei, Y. Germline stem cells. *Cold Spring Harbor Perspectives in Biology* **3** (2011). 10.1101/cshperspect.a002642 PMC3220357 21791699

[7] Fuller MT, Spradling AC (2007). Male and female Drosophila germline stem cells: two versions of immortality. Science.

[8] Matunis EL, Stine RR, Cuevas MD (2012). Recent advances in Drosophila male germline stem cell biology. Spermatogenesis.

[9] Schulz C, Wood CG, Jones DL, Tazuke SI, Fuller MT (2002). Signaling from germ cells mediated by the rhomboid homolog stet organizes encapsulation by somatic support cells. Development.

[10] Knippschild U (2005). The casein kinase 1 family: participation in multiple cellular processes in eukaryotes. Cellular Signalling.

[11] Castrillon DH (1993). Toward a molecular genetic analysis of spermatogenesis in Drosophila melanogaster: characterization of male-sterile mutants generated by single P element mutagenesis. Genetics.

[12] Hummel T, Attix S, Gunning D, Zipursky SL (2002). Temporal control of glial cell migration in the Drosophila eye requires gilgamesh, hedgehog, and eye specification genes. Neuron.

[13] Schulz C (2004). A Misexpression Screen Reveals Effects of bag-of-marbles and TGFβ Class Signaling on the Drosophila Male Germ-Line Stem Cell Lineage. Genetics.

[14] Davidson G (2005). Casein kinase 1 gamma couples Wnt receptor activation to cytoplasmic signal transduction. Nature.

[15] Nerusheva OO, Dorogova NV, Gubanova NV, Yudina OS, Omelyanchuk LV (2009). A GFP trap study uncovers the functions of Gilgamesh protein kinase in Drosophila melanogaster spermatogenesis. Cell Biology International.

[16] Tan Y, Yu D, Pletting J, Davis RL (2010). Gilgamesh is required for rutabaga-independent olfactory learning in Drosophila. Neuron.

[17] Kawase E, Wong MD, Ding BC, Xie T (2004). Gbb/Bmp signaling is essential for maintaining germline stem cells and for repressing bam transcription in the Drosophila testis. Development.

[18] Sheng XR (2009). Jak-STAT regulation of male germline stem cell establishment during Drosophila embryogenesis. Developmental Biology.

[19] Wang H (2006). Rap-GEF Signaling Controls Stem Cell Anchoring to Their Niche through Regulating DE-Cadherin-Mediated Cell Adhesion in the Drosophila Testis. Developmental cell.

[20] Bustin SA (2002). Quantification of mRNA using real-time reverse transcription PCR (RT-PCR): trends and problems. Journal of Molecular Endocrinology.

[21] Lhocine N (2008). PIMS modulates immune tolerance by negatively regulating Drosophila innate immune signaling. Cell Host & Microbe.

[22] Gupta RK, Prasad S (2015). Optimization of multiplex RT-PCR for M1, M23, and M23X splice variants of AQP4 and β-actin transcripts in Dalton’s lymphoma mouse tissues. Turkish Journal of Biology.

[23] Chen D (2009). Effete-mediated degradation of Cyclin A is essential for the maintenance of germline stem cells in Drosophila. Development.

[24] Tran J, Brenner TJ, Dinardo S (2000). Somatic control over the germline stem cell lineage during Drosophila spermatogenesis. Nature.

[25] Kiger AA, Jones DL, Schulz C, Rogers MB, Fuller MT (2001). Stem cell self-renewal specified by JAK-STAT activation in response to a support cell cue. Science.

[26] Ferrandon D (1998). A drosomycin-GFP reporter transgene reveals a local immune response in Drosophila that is not dependent on the Toll pathway. The EMBO Journal.

[27] Xu T, Rubin GM (1993). Analysis of genetic mosaics in developing and adult Drosophila tissues. Development.

[28] Xie T, Spradling AC (1998). decapentaplegic Is Essential for the Maintenance and Division of Germline Stem Cells in the Drosophila Ovary. Cell.

[29] Tulina N, Matunis E (2002). Control of stem cell self-renewal in Drosophila spermatogenesis by JAK-STAT signaling. Science.

[30] Rørth P (1998). Gal4 in the Drosophila female germline. Mechanisms of Development.

[31] Van DM, Williamson AL, Lehmann R (1998). Regulation of zygotic gene expression in Drosophila primordial germ cells. Current Biology.

[32] Chen D, Mckearin DM (2003). A discrete transcriptional silencer in the bam gene determines asymmetric division of the Drosophila germline stem cell. Development.

[33] Tsuneizumi K (1997). Daughters against dpp modulates dpp organizing activity in Drosophila wing development. Nature.

[34] Inoue H (1998). Interplay of signal mediators of decapentaplegic (Dpp): molecular characterization of mothers against dpp, Medea, and daughters against dpp. Molecular Biology of the Cell.

[35] Ohlstein B, Mckearin D (1997). Ectopic expression of the Drosophila Bam protein eliminates oogenic germline stem cells. Development.

[36] Nerusheva OO, Dorogova NV, Gubanova NV, Omel’Yanchuk LV (2008). The role of Gilgamesh protein kinase in Drosophila melanogaster spermatogenesis. Russian Journal of Genetics.

[37] Spradling AC, Rubin GM (1982). Transposition of cloned P elements into Drosophila germ line chromosomes. Science.

[38] Chen D, Mckearin D (2003). Dpp signaling silences bam transcription directly to establish asymmetric divisions of germline stem cells. Current Biology.

[39] Jiang X (2008). Otefin, a nuclear membrane protein, determines the fate of germline stem cells in Drosophila via interaction with Smad complexes. Developmental cell.

