# Supplementary material for: Gilgamesh is required for the maintenance of germline stem cells in Drosophila testis
Source: Sci Rep. 2017 Jul 18;7:5737. doi: 10.1038/s41598-017-05975-w (PMC5515886; doi:10.1038/s41598-017-05975-w)
Supplement: Supplementary file 1 — Gilgamesh is required for the maintenance of germline stem cells in Drosophila testis [file 41598_2017_5975_MOESM1_ESM.pdf]

Title: Gilgamesh is required for the maintenance of germline stem cells in *Drosophila* testis

Authors: Dongsheng Chen[1,2,3,*], Xiangxiang Zhu[1,3] , Lijuan Zhou[1,3], Jian Wang[1], Xiaoqian Tao[1], Shuang Wang[1], Fuling Sun[1], Xianzhao Kan[1,2], Zhengqi Han[1], Yuelin Gu[1]

1. Provincial Key Laboratory of the Conservation and Exploitation Research of Biological Resources in Anhui, College of Life Sciences, Anhui Normal University, Wuhu 241000, China.
2. The Institute of Bioinformatics, College of Life Sciences, Anhui Normal University, Wuhu 241000, China.
3. These authors contributed equally to this work.

* Corresponding author: Dongsheng Chen, Provincial Key Laboratory of the Conservation and Exploitation Research of Biological Resources in Anhui, College of Life Sciences, Anhui Normal University, 1 Beijing East Road, Wuhu 241000, China.

TEL:86-133-9953-5126; E-mail: cds2001@ahnu.edu.cn

Supplementary information

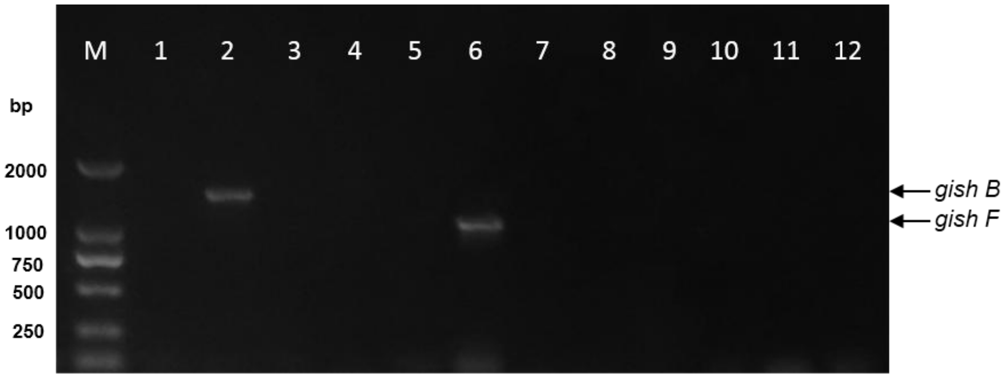

Figure S1. Detection of *gish* splice variants in fly testis by RT-PCR. Agarose gel showing the products of the primer pairs mentioned in S1 Tab. Lanes 1-12 representthe primer pairs that can amplify the *gish* splice variants such as A, B, C, D/L, E, F, G, H, I, J, K and M, respectively. The PCR products (~1400bp size in lane 2, ~1000bp size in lane 6) were detected in agarose gel, which demonstrates that *gishB* (lane 2) and *F* (lane 6) are specifically expressed in fly testis. Lane M denotes DNA ladder.

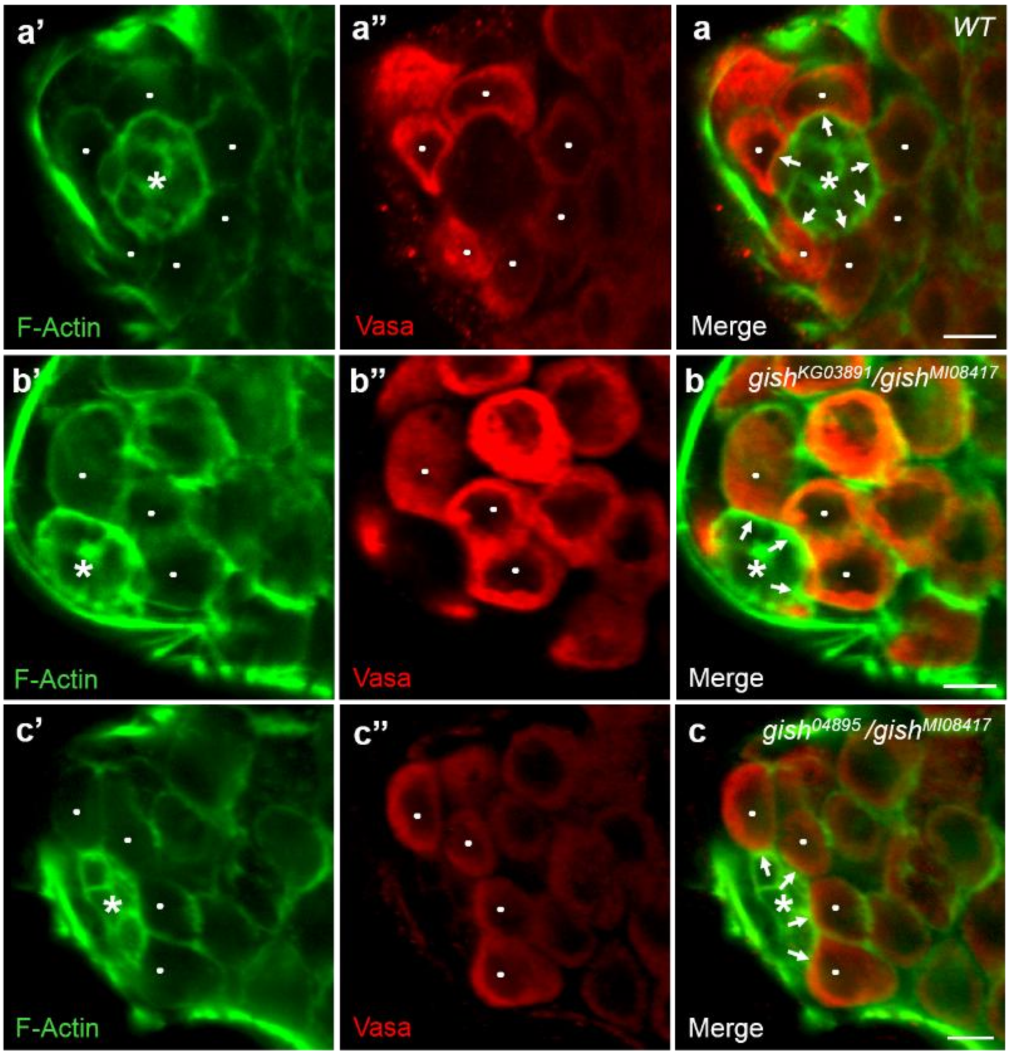

Figure S2. The mutation of *gish* doesn't affect GSCs' adhesion to the hub. Testes from 14-day-old male flies were stained with FITC-conjugated Phalloidin (green, hub cells and germ cells) and anti-Vasa antibody (red, germ cells). The hub was noted by asterisk (green), GSCs were indicated by white dots (red). The boundary of GSCs adhesion to hub were indicated by arrows. (a'-a) Testis from wild-type fly. (b'-b) Testis from $gish^{KG03891}/gish^{MI08417}$ fly. (c'-c) Testis from $gish^{04895}/gish^{MI08417}$ fly. There were no difference in cell-cell adhesions (GSC and hub cell) between *gish* mutants and wild-type testes. Scale bars: 5μm.

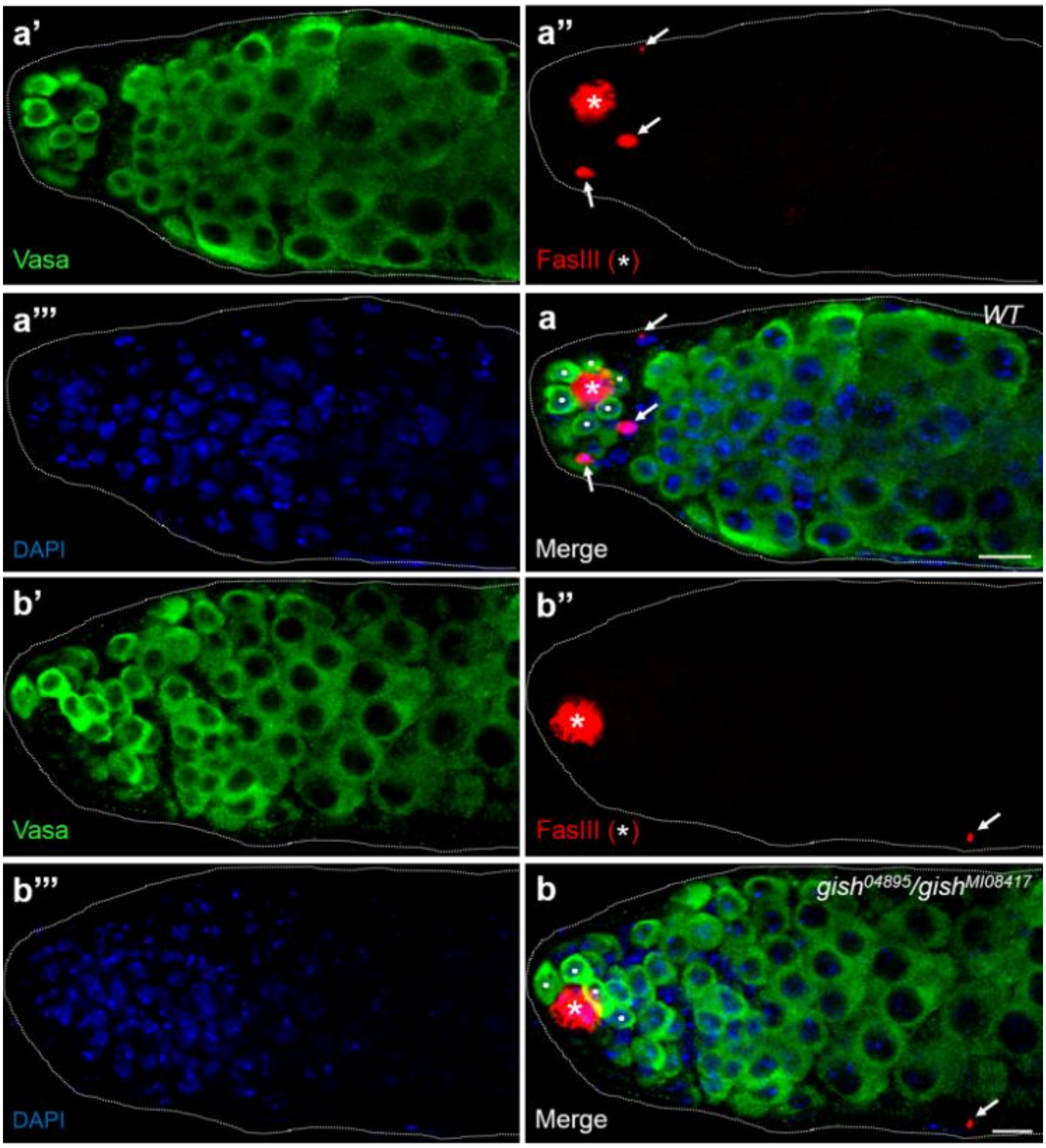

Figure S3. Loss of GSC in *gish* mutant is not caused by cell death. Testes from male flies were labeled by TUNEL (red, indicated by arrows), stained with anti-Vasa antibody (green, germ cells) and anti-Fas III antibody to label the hub (red, asterisk). Germline stem cells (GSCs) were indicated by white dots. The outlines of testes were drawn bybroken lines. (a'-a) Testis from wild-type fly. (b'-b) Testis from $gish^{04895}$/ $gish^{MI08417}$ fly. Cell death was not detected in GSCs (indicated by white dots), but in somatic cells (indicated by arrows, negative for anti-Vasa staining). Scale bars: 10μm.

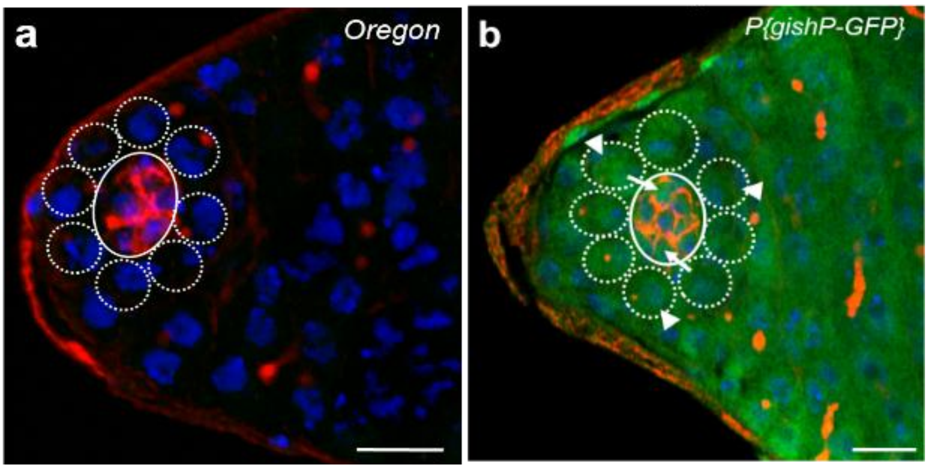

Figure S4. the gene *gish* expresses broadly in adult fly testis. (a) Testis from *Oregon* fly (*wild-type*). (b) Testis bearing a transgene P{*gishP-GFP*}. (a-b) Testes were labeled by with anti-GFP (green), anti-Fas III antibody (red, marking the hub), anti-Hts antibody (red, labeling the germ cells) and DAPI (blue). The gene *gish* expresses (green) both in GSCs (broken lines, arrowhead) and in hub cells (a circle, arrow). Scale bars: 10μm.

**The splicing variants for *gish***	**The sequences of primer pairs**	**The length of RT-PCR products (bp)**
gish A	Forward: CCAACCAGATGTGCAATAAAAGTG	800
	Reverse: TAATTGTATAGCTATCAGAAGTACGC	
gish B	Forward: ATGCAGCGACGAGAACGGCAAGC	1407
	Reverse: TCATTTTTGGCGCGTCGATTTC	
gish C	Forward: CCAACCAGATGTGCAATAAAAGTG	810
	Reverse: AGTTGTTTCGCTATCATCAATACAG	
gish L&D	Forward: GCAGATTCGTTTTTTAGTCCCAAATC	2640
	Reverse: TTACAACGGAAGTGTCGGTGTG	
gish E	Forward: ATGCAGCGACGAGAACGGCAAGC	500
	Reverse: TAATTGTATAGCTATCAGAAGTACGC	
gish F	Forward: ATGCAGCGACGAGAACGGCAAGC	1077
	Reverse: TAATTGCTTGCAATTCACTATGTCTC	
gish G	Forward: ATGTTGCGCAAATATTTATAC	1410
	Reverse: TCATTTTTGGCGCGTCGATTTC	
gish H	Forward: CGGACGCTAAATCCTGCAGA	1607
	Reverse: TCATTTTTGGCGCGTCGATTTC	
gish I	Forward: ATGCAGCGACGAGAACGGCAAGC	1370
	Reverse: CTGGAATATAAGGAACCATTT	
gish J	Forward: ATGCAGCGACGAGAACGGCAAGC	1300
	Reverse: CTGGAGTATTATATATTCTTCATTCTC	
gish K	Forward: GTCGGCGAATGTGTTGCTTTGGTG	1205
	Reverse: CTGGAATATAAGGAACCATTT	
gish M	Forward: ATGCAGCGACGAGAACGGCAAGC	2400
	Reverse: AAGTGATTGATCTTTGCGGAAACTTG	

Table S1. Primers for detecting of *gish* mRNA splicing variants in *Drosophila* testis.

**Genotype**	**The average number of GSCs in fly testis in different day after eclosion (Mean ± SD)**	
	**Day 1**	**Day 20**
gish^{04895}/*gish*^{KG03891}	6.7 ±1.4 (n=67)	3.7 ±1.0 (n=64)
P{*gishP-gishF*}; *gish*^{04895}/*gish*^{KG03891}	7.9 ±1.2 (n=62)	7.5 ±1.0 (n=65) *
gish^{04895} /*gish*^{MI08417}	6.8 ±1.3 (n=64)	3.5 ±1.0 (n=65)
P{*gishP-gishF*}; *gish*^{04895} /*gish*^{MI08417}	7.7 ±1.4 (n=62)	7.5 ±1.2 (n=65) *
gish^{KG03891}/*gish*^{MI08417}	6.9 ±1.0 (n=66)	3.3 ±1.3 (n=67)
P{*gishP-gishF*}; *gish*^{KG03891}/*gish*^{MI08417}	7.8 ±1.0 (n=64)	7.5 ±1.2 (n=64) *

Table S2. The rescue assay of *gish* mutants by the transgenic line of P{*gishP-gishF*}. SD, standard deviation. n, Number of testes examined. $^{*}P < 0.01$, unpaired *t*-test, compared with the corresponding *gish* mutant at day 20.

**Genotype**	**The percentage of apoptotic GSCs between wild type and *gish* mutant fly testes**
Oregon-R	0.45% (N=220)
gish^{KG03891}/*gish*^{MI08417}	0.40% (N=246)
gish^{04895} /*gish*^{MI08417}	0.38% (N=237)

Table S3. The analysis of the cell death in *gish* mutant testes at day 5 after eclosion. N, the total number of GSCs examined.

**Genotype**	**The average number of GSCs in fly testis in different day after eclosion (Mean ± SD)**	
	**Day 1**	**Day 20**
gish^{04895}/*gish*^{04895}	6.7 ±1.4 (n=67)	3.6 ±1.1 (n=66)
P{*nosP-gishF*}; *gish*^{04895}/*gish*^{04895}	7.7 ±1.3 (n=65)	7.5 ±1.1 (n=66) *
gish ^{KG03891} /*gish*^{MI08417}	6.8 ±1.0 (n=70)	3.3 ±1.3 (n=69)
P{*nosP-gishF*}; *gish* ^{KG03891} /*gish*^{MI08417}	7.6 ±1.1 (n=64)	7.4 ±1.1 (n=67) *

Table S4. The rescue asssay of *gish* mutants by the transgenic line of P{*nosP-gishF*}. SD, standard deviation. n, Number of testes examined. $^{*}P < 0.01$, unpaired *t*-test, compared with the corresponding *gish* mutant at day 20.

Genotype	The average number of GSCs in fly testis in different day after eclosion (Mean ± SD)	
	Day 1	Day 20
gish^{04895}/*gish*^{MI08417}	6.8 ±1.1 (n=69)	3.6 ±1.2 (n=68)
P{*c587-gal4*};P{*UASp-gishF*}; *gish*^{04895}/ *gish*^{MI08417}	6.9 ±1.1 (n=65)	3.5 ±1.2 (n=66) *
gish^{KG03891}/*gish*^{MI08417}	6.8 ±1.2 (n=70)	3.4 ±1.2 (n=69)
P{*c587-gal4*};P{*UASp-gishF*}; *gish*^{KG03891}/*gish*^{MI08417}	6.8 ±1.1 (n=66)	3.3 ±1.0 (n=68) *

Table S5. The rescue asssay of *gish* mutants by combining the transgenic lines of P{*c587-gal4*} and P{*UASp-gishF*}. SD, standard deviation; n, Number of testes examined. $^{*}P > 0.05$, unpaired *t*-test, compared with the corresponding *gish* mutant at day 20.

Genotype	The average number of GSCs in fly testis in different day after eclosion (Mean ± SD)	
	Day 1	Day 20
gish^{04895}/*gish*^{KG03891}	6.7 ±1.4 (n=65)	3.6 ±1.1 (n=66)
P{*UASp-gishF*}/P{*nos-gal4*}; *gish*^{04895}/*gish*^{KG03891}	7.7 ±1.3 (n=66)	7.5 ±1.1 (n=67) *
gish^{04895} /*gish*^{MI08417}	6.8 ±1.0 (n=70)	3.4 ±1.2 (n=69)
P{*UASp-gishF*}/P{*nos-gal4*}; *gish*^{04895}/*gish*^{MI08417}	7.6 ±1.1 (n=64)	7.4 ±1.1 (n=67) *
gish^{KG03891}/*gish*^{MI08417}	6.8 ±1.0 (n=69)	3.3 ±1.3 (n=69)
P{*UASp-gishF*}/P{*nos-gal4*}; *gish*^{KG03891}/*gish*^{MI08417}	7.5 ±1.1 (n=67)	7.4 ±1.1 (n=69) *

Table S6. The rescue asssay of *gish* mutants by combining the transgenic lines of P{*nos-gal4*}and P{*UASp-gishF*}. SD, standard deviation. n, Number of testes examined. $^{*}P < 0.01$, unpaired *t*-test, compared with the corresponding *gish* mutant at day 20.
